# Inhibitory effects of adenovirus mediated tandem expression of RhoA and RhoC shRNAs in HCT116 cells

**DOI:** 10.1186/1756-9966-28-52

**Published:** 2009-04-18

**Authors:** Xiang-ping Liu, Hai-bo Wang, Kun Yang, Ai-hua Sui, Qiang Shi, Shen Qu

**Affiliations:** 1 Department of Biochemistry and Molecular Biology, School of Basic Medical Sciences, Tongji Medical College, Huazhong University of Science and Technology, Wuhan 430030, PR China; 2 Central Laboratory of Molecular Biology, Affiliated Hospital of Qingdao University Medical College, Qingdao, 266003, PR China; 3 Department of General Surgery, Affiliated Hospital of Qingdao University Medical College, Qingdao, 266003, PR China

## Abstract

**Background:**

RhoA and RhoC are deregulated by over expression in many human tumors, including colorectal cancer. Some reports show that they play a pivotal role in the carcinogenesis, tumor development and infiltration metastasis. In this study, for the first time we constructed recombinant adenovirus to investigate the inhibitory effects of RhoA and RhoC shRNAs in tandem expression on the cell proliferation and invasion of colorectal cancer HCT116 cells.

**Methods:**

The recombinant adenovirus carrying RhoA and RhoC shRNAs in tandem expression was transfected into HCT116. The mRNA transcription and protein expressions of RhoA and RhoC were examined by RT-FQPCR and Western blot respectively. Cellular proliferation inhibitory activity was determined by methyl thiazolyl tetrazolium (MTT) assay and invasive and migrating potential was detected through in vitro Matrigel coated invasion and migration assay.

**Results:**

Both mRNA and proteins Levels of RhoA and RhoC were significantly reduced in HCT116 cells transfected with Ad-A1+A2+C1+C2 than those in Ad-HK group and control one. The relative RhoA and RhoC mRNA transcriptions were decreased to 40% and 36% (P < 0.05), while proteins expression reducing 42% and 35%, respectively (P < 0.05). Growth curves analysis showed that alive cell number in the Ad-A1+A2+C1+C2 group was lower than others in the third to sixth day and transwell chamber analysis showed that migration/invasion activity was significantly suppressed in Ad-A1+A2+C1+C2 group.

**Conclusion:**

Our results indicate recombinant adenovirus carrying RhoA and RhoC shRNAs in tandem expression may inhibit the growth and invasion of HCT116 cells. Application of such vector to inhibit one or more genes may be a new method to cancer therapy.

## Background

The Rho family, a member of the Ras superfamily of low-molecular-weight GTP-binding proteins, contains Rho (e.g., RhoA, B, C), Rac and Cdc42 proteins [1,2] Previous studies have shown that Rho proteins become deregulated by over expression in tumours. In some cases, this deregulation correlates with disease progression [3]. Despite the high homology of different Rho isoforms (RhoA, RhoB and RhoC), their physiological roles are distinct [4]. The role of RhoB in these processes appears to be more divergent, whereas RhoA and RhoC proteins have been shown to have a positive role in proliferation and malignant transformation [5,6]. Moreover, elevated RhoC expression has been found to correlate with poor outcome in whites with colorectal carcinoma and may be used as a prognostic marker of colorectal carcinoma. Increased levels of RhoA expression was observed in Asian patients with colorectal carcinoma. Therefore, specific inhibiting the functions of RhoA and RhoC are predicted to be of great therapeutic benefits. Recently, it has been demonstrated that interfering the expression of RhoA and RhoC using small interfering RNA (siRNA) approaches inhibited the proliferation and invasion of gastric cancer cells [7].

In this study, for the first time we constructed adenovirus vector carrying RhoA and RhoC shRNAs in tandem expression and investigated the inhibitory effects of recombinant adenovirus on the cell proliferation and invasion of colorectal cancer HCT116 cells. We showed that a significant reduction in RhoA and RhoC expression could markedly inhibit the invasion and migration potentials of colorectal cancer cells. Thus, our results provide new evidence of the potential use of one more gene-targeted RNAi as a novel way to reduce tumor progression of colorectal cancer.

## Methods

### Cell culture

The human colon cancer cell line HCT116 was purchased from China Centre for Type Culture Collection, Chinese Academy of Sciences. The cells were grown in McCoy's 5A medium, Modified (Sigma), supplemented with 10% of fetal bovine serum (Hyclone, USA) at 37°C in a humidified atmosphere of 5% CO_2_. Cells were always detached using Trypsin-EDTA and subcultured at 1.5 × 10^5 ^cells per well into six-well tissue culture plates for transfection.

### Cell transfection with adenovirus vectors

Four kinds of oligonucleotide sequences that specifically knock out human RhoA (NM_001664) and RhoC (NM_175744) were selected [8]. The oligonucleotide sequence was as follows: A1: GAAGGCAGAGATATGGCAA, A2: GAAGGATCTTCGGAATGAT, C1: CTATATTGCGGACATTGAG, C2: AACATTCCTGAGAAGTGGA. Scrambled control: GACTTCATAAGGCGCATGC. 4 pairs shRNA (A1, A2, C1 and C2) were then cloned into the vector pGenesil-2 (with hU6, mU6, h7SK and hH1 promoters respectively) by repeated excision and ligation successively. The recombinant adenovirus was generated by Jingsai biological CO. LTD, Wuhan, China. The particle titers of the adenoviral stocks were 1 × 10^9 ^plaque-forming units per milliliter (pfu/mL). Adenovirus vectors expressing RhoA and RhoC (Ad-A1+A2+C1+C2, A1+A2+C1+C2 in tandem), green fluorescent protein (Ad-GFP) or negative control (Ad-HK) were used to transfect HCT116 cells. Transfection efficiency was detected directly by testing the expression ratio of green fluorescent protein. When multiplicity of infection (MOI) was 10, HCT116 cells were co-cultured with Ad-A1+A2+C1+C2 or Ad-HK. The cells were collected after being transfected for 48 h. Untreated cell was used as control.

### Reverse transcription-fluoresencent quantitative polymerase chain reaction (FQ-PCR)

Total RNA was extracted from each sample using Trizol (Invitrogen, Gaithersburg, MD) and reversely transcripted into cDNA using the PrimeScript RT-PCR kit (TaKaRa Bio Inc., Shiga, Japan) according to the manufacturer's instructions. The primers for the human RhoA gene were: sense 5'-CGGGAGCTAGCCAAGATGAAG-3', antisense 5'-CCTTGCAGAGCAGCTCTCGTA-3', fluorescent probe 5'-FAM-AGAGATATGGCAAACAGGATTGGCG-TAMRA-3', and the amplicon size is 158 base pairs (bps). The primers for the human RhoC gene were: sense 5'-CCTCATGTGCTTCTCCATCGA-3', antisense 5'-CTCGTCTTGCCTCAGGTCCTT-3', fluorescent probe 5'-FAM-TCTGCCCCAACGTGCCCATCAT-TAMRA-3', and the amplicon size is 136 bps. The GAPDH was used as the internal control with the specific primers: sense 5'-CTTAGCACCCCTGGCCAAG-3', antisense 5'-GATGTTCTGGAGAGCCCCG-3', fluorescent probe 5'-FAM-CATGCCATCACTGCCACCCAGAAGA-TAMRA-3', and the amplicon size is 150 bps. Primers and fluorescent probes were synthesized by Shanghai Sangon Biological Engineering Technology & Services Co., Ltd. (Shanghai, China). The levels of RhoA, RhoC and control GAPDH mRNA transcripts were determined by the QRT-PCR in ABI7500 real time thermal cycler (Applied Biosystems, Foster City, CA). The PCR reactions in duplicate were subjected to an initial denaturation at 95°C for 10 seconds, followed by 40 cycles of denaturation at 95°C for 5 seconds, annealing and extension at 60°C for 45 seconds. The value of threshold cycle (CT) for each reaction was recorded.

### Western blot analysis

Cell samples were lysed in ice-cold lysis buffer (Beyotime, China) with 1% PMSF (Phenylmethylsulfonyl fluoride) for half an hour, then centrifuged at 10,000 g for 20 min at 4°C and the protein concentration of the resulting supernatant was determined by the bicinchoninic acid (BCA) protein assay kit (Beyotime, China). Proteins (50 μg) were separated by 12% SDS-PAGE electrophoresis and subsequently transferred to PVDF membranes. Membranes were blocked with 5% nonfat dry milk in TBS/Tween 20 (0.05%, v/v) for 2 h at room temperature and incubated overnight at 4°C with primary antibodies directing against RhoA (Santa Cruz), RhoC (Santa Cruz) and GAPDH. The blots were washed and incubated with the horseradish peroxidase-conjugated secondary antibody (DakoCytomation), and developed with a chemiluminescent substrate, ECL Plus. An autoradiograph was obtained, and protein levels were measured using a Fluors scanner and Quantity One software for analysis (Bio-Rad). Assays were done in triplicate for each experiment, and each experiment was repeated three times.

### MTT assay

For measurement of cell proliferation rate, the collected cells, which were transfected with Ad-A1+A2+C1+C2 or Ad-HK for 48 h, were plated into 96-well plates in 1 × 10^3 ^cells/100 μl medium/well. 10 μl of MTT solution (Amresco) was added into each well daily from the 2nd to 7th day, and plates were incubated for 4 h at 37°C. Then 150 μl DMSO was added to dissolve formazan. Absorbance values (A) were measured at a wavelength of 490 nm with a microplate reader. Results were expressed as mean value ± SEM and surviving rate was calculated as the follows: Surviving rate = A_490 _of experiment/A_490 _of control × l00%. Assay was done in six wells, and each experiment was repeated three times.

### In vitro matrigel invasion assay

In vitro Matrigel invasion assay was performed by using a 24-well millicell inserts (BD Biosciences) with polycarbonate filters (pore size, 8 μm). The upper side of polycarbonate filter was coated with matrigel (50 μg/ml, BD Biosciences). The chambers were incubated at 37°C with 5% CO2 for 2 h to allow the matrix to form a continuous thin layer. Then the cells transfected with Ad-A1+A2+C1+C2 or Ad-HK and control ones were harvested and 4 × 10^5 ^cells in 200 μl of 0.1% bovine serum albumin were placed in the upper chamber. The lower chamber was filled with 10% serum-medium (700 μl). Cells were cultured for 22 h at 37°C in 5% CO2. Cells on the upper surface of the filter were removed using a cotton swab. Cells invading through the Matrigel and filter to the lower surface were fixed with 4% neutral-buffered formalin and stained in 0.01% crystal violet solution. The cell numbers in five fields (up, down, median, left, right. ×200) were counted for each chamber, and the average value was calculated. Assays were done in triplicate for each experiment, and each experiment was repeated three times.

### In vitro cell migration assay

This migration assay was to measure cell migration through an 8.0-μm pored membrane in a 24-well millicell inserts (BD Biosciences). The lower chamber was filled with 10% serum-medium (700 μl). 4 × 10^5 ^cells in 200 μl medium supplemented with 10% FBS were placed in the upper chamber. After 16h-incubation, the number of migrated cells (lower side of the membrane) was counted as described above.

### Statistical analysis

Statistical analyses were performed using SPSS statistical software (SPSS Inc., Chicago, Illinois). Data were shown by mean value ± SEM. Differences between two groups were assessed using a t test. A P value less than 0.05 was considered statistically significant.

## Results

### Transfection of HCT116 with adenovirus

**Figure 1:**
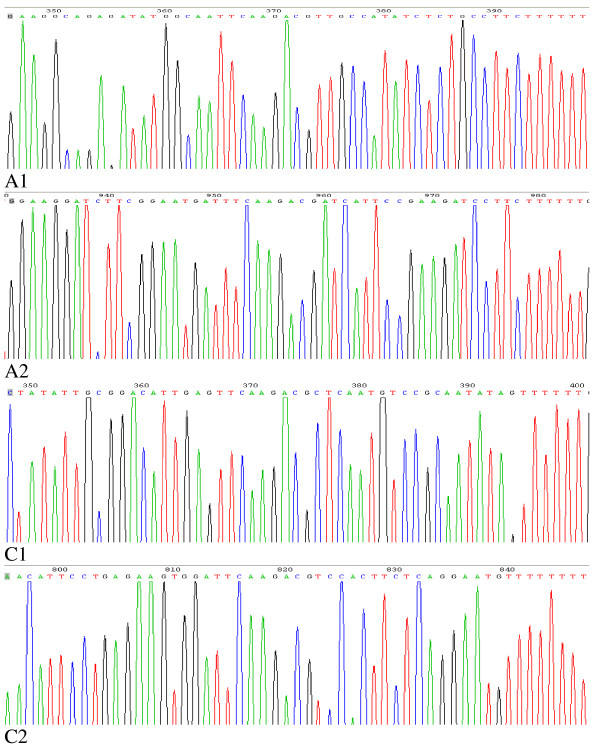
**shows the sequencing histograms of A1, A2, C1 and C2 of Ad-A1+A2+C1+C2**. They all contain the sense +loop (TTCAAGACG)+antisense.

**Figure 2:**
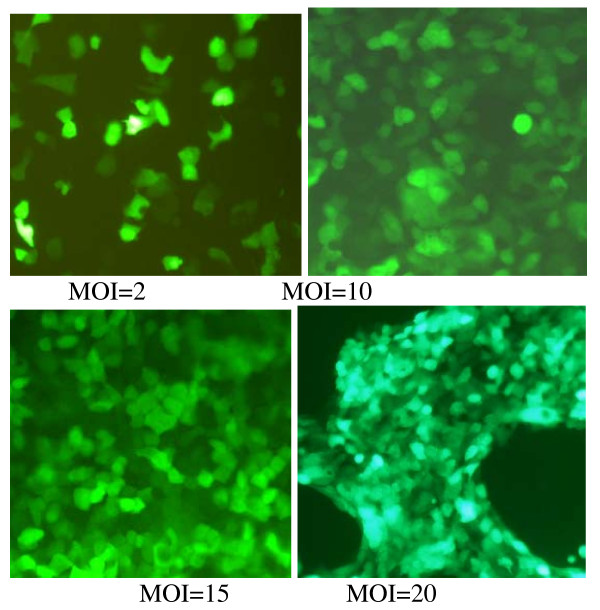
**displays the expression of GFP in HCT116 cells 48 h after transfected by Ad-GFP with different MOIs under fluorescent microscope at 200× magnification**. The number of GFP-expressing cells increases in a MOI-dependent manner. When the MOI is more than 20, the infected cells still display bright green fluorescence, but their morphologies changes dramatically with less vigorously growing.

Through sequence analysis, the Ad-A1+A2+C1+C2 vector was identified to be constructed successfully (Fig. 1). To assess the efficiency of adenoviral transduction, human HCT116 cells were plated at a density of 1.5 × 10^5 ^cells/well into 24-well plates and infected with Ad-GFP at various multiplicities of infection (MOIs) 24 h after seeding. After 48 h, GFP-expressing cells were detected by fluorescence microscopy (Olympus, Japan). We found that HCT116 cells exhibited high adenoviral transduction efficiency, because more than 95% cells were infected with Ad-GFP at a dose of MOI 10. We also found that the number of GFP-expressing cells increased in a MOI-dependent manner (Fig. 2), but cytotoxicity was gradually achieved at higher dose of virus (MOI > 20).

### Silencing of specific genes and proteins in HCT116

**Figure 3:**
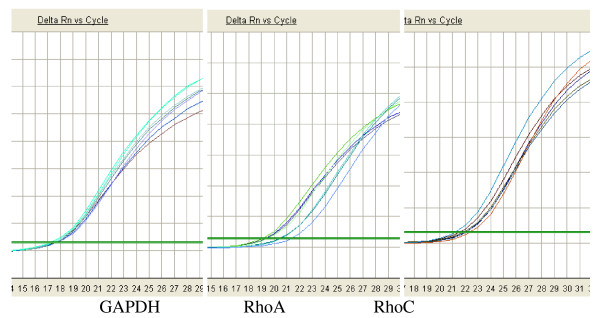
**shows the amplification curve of GAPDH, RhoA and RhoC**. They all exhibit standard S shape, suggesting a good amplification efficiency and linear relationship.

**Figure 4:**
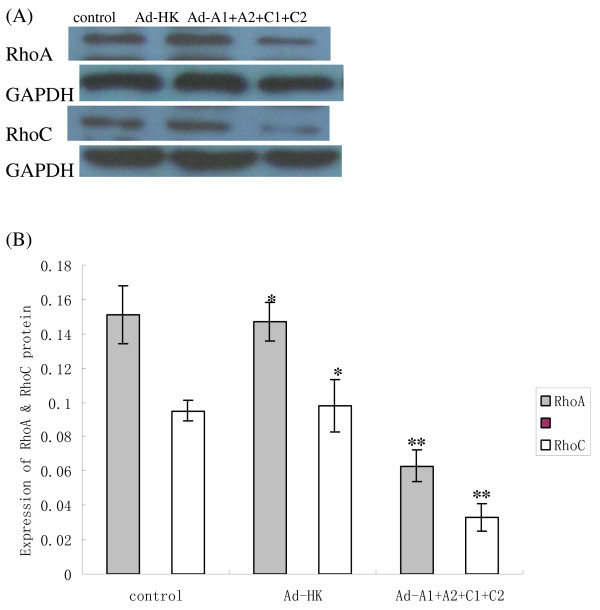
**indicates protein levels in HCT116 cells**. The RhoA and RhoC proteins from cells infected with Ad-A1+A2+C1+C2 were significantly weaker than those from control cells or from cells infected with Ad-HK. GAPDH is used as a loading control (A). The graph (B) compares scanning signal intensity of RhoA and RhoC expression by Imagel software. *P > 0.05, no significantly difference between the cells treated with Ad-HK and the control cells. **P < 0.05, compared with other groups.

48 hours after transfection of Ad-A1+A2+C1+C2 or Ad-HK to HCT116, we analyzed the expression of RhoA and RhoC in mRNA and protein level in HCT116 cells using real-time FQ-PCR [9] and Western blot assay respectively. The ΔCT (CT_Target _- CT_GAPDH_) values for RhoA and RhoC mRNA for cells infected with Ad-A1+A2+C1+C2 were significantly higher than those for cells that were infected with Ad-HK or for the control cells (Fig. 3, Table 1). The relative RhoA and RhoC mRNA expression to the control cells were only about 40% and 36%, respectively, which demonstrated a significantly reduced expression of RhoA and RhoC mRNA (P < 0.05). However, there was no significant difference between the cells treated with Ad-HK and the control ones (P > 0.05). As shown in Fig. 4, RhoA and RhoC protein expression was similar to the results of FQ-PCR. The scanning signal intensity of RhoA and RhoC proteins for cells infected with Ad-A1+A2+C1+C2 were significantly weaker than those of control cells or cells infected with Ad-HK (P < 0.05). The relative RhoA and RhoC protein expression of cells infected with Ad-A1+A2+C1+C2 to the control cells were only about 42% and 35%, respectively (P < 0.05).

**Table 1:** Expression of RhoA and RhoC mRNA in human HCT116 cells (mean ± SEM)

	**RhoA**		**RhoC**	
**Groups**	**ΔΔCT**	**Rel. to control^a^**	**ΔΔCT**	**Rel. to control^a^**
Control	0 ± 0.17	1 (0.88–1.13)	0 ± 0.11	1 (0.93–1.08)
Ad-HK	0.11 ± 0.09	0.93 (0.87–0.99)	0.13 ± 0.10	0.91 (0.85–0.98)
Ad-A1+A2+C1+C2	1.32 ± 0.22	0.40 (0.34–0.47)	1.48 ± 0.16	0.36 (0.32–0.40)

a. Data are expressed as the mean 2^-ΔΔCT ^(range).

### Effects of RhoA and RhoC specific shRNA on cell proliferation activity

**Figure 5:**
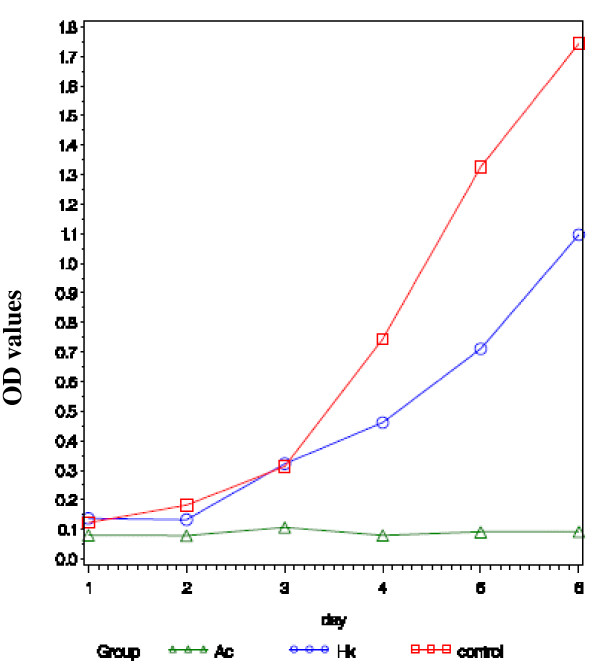
**displays the growth curve according to the values of 490 nm wavelength light absorption in the three groups**. In the third to sixth day, significant difference as exhibited in cell growth inhibition in Ad-A1+A2+C1+C2 group. But there is a slight difference between the control cell and the cells infected with Ad-HK.

To assess the proliferation activity of tumor cell is important in its invasion and metastasis. Collected cells were seeded onto 96-well microplates and cellular growths were determined by a continuous 6-day MTT assay. Growth curve was plotted according to these OD value alterations of MTT assay. The difference in cell growth inhibition rate between the HCT116 cells infected with Ad-A1+A2+C1+C2 and the other two groups was not statistically significant in the first 2 days. However, in the third to sixth day, significant differences were found (Fig. 5), but no significant difference between the control cells and the cells infected with Ad-HK. The results showed that knockdown of RhoA and RhoC in the HCT116 cells by shRNA could change the cell proliferation activity in vitro.

### Invasion and migration power assay in vitro

**Figure 6:**
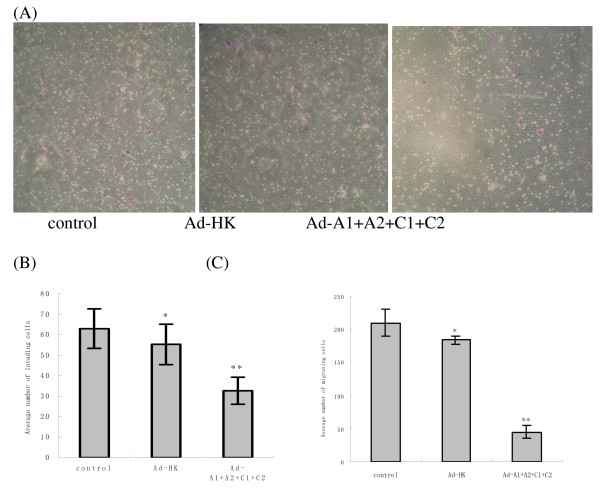
**indicates that silencing of RhoA and RhoC may inhibit the invasion and migration of HCT116 cells**. The number of invading cells was determined by counting the cells stained with 0.01% crystal violet solution in the lower side of the membrane (A). The graphs (B, C) compare the numbers of transmembrane cells in invasion and migration experiments. Data represent the mean value ± SEM of three independent experiments. *P > 0.05, no significantly difference between the cells treated with Ad-HK and the control cells. **P < 0.05, compared with other groups.

After 22 h incubation, the control HCT116 cells showed stronger invasion activities compared with the ones infected with Ad-A1+A2+C1+C2 group (88 versus 38) (Fig. 6). The differences between the control and Ad-HK group had no statistical significance. Moreover, the HCT116 cells in Ad-A1+A2+C1+C2 group displayed a significantly lower transmembrane migration activity as compared to those in Ad-HK group and in control HCT116 cells. These findings suggest that RhoA and RhoC expression level seems to be closely associated with the enhanced invasion and migration in HCT116 cell lines.

## Discussion

Rho GTPases act as molecular switches to control signal transduction pathways by cycling between a GDP-bound, inactive form and a GTP-bound, active form. Their best-characterized function is in the regulation of actin dynamics. They not only regulate the organization of actin filament system, but also modulate cell motility, proliferation, apoptosis, cell cycle progression, and invasion and metastasis of malignant tumor cells [10,11]. Cell migration is an essential process in multicellular organisms but it's a critical step in tumor invasion and metastasis. Understanding of this process may lead to appropriate therapies for cancer [12,13]. Recent accumulating evidences have shown that RhoA and RhoC are over-expressed in many kinds of cancers, and they may play important roles in initiation and progression of cancers [3,5,14,15].

Despite the high homology of RhoA and RhoC, RhoA has been shown to regulate the activities of multiple transcription factors, most of which are implicated in the cancer progression [16] by modulating cancer cell adhesion, contraction, movement, release of cellular adhesion, degradation of extra-cellular matrix, and invasion into blood or lymph vessels [17,18]. RhoC also contributes to tumor development, especially to invasion and metastasis of cancer cells [19,20]. Furthermore, Faried A. and colleagues identified that RhoA promoted tumour growth more than RhoC, while RhoC induced distant metastasis in comparison to RhoA [21]. These findings are alike to those of Clark and colleagues, who showed that RhoC had better motogen than RhoA when expressed in melanoma and that RhoC over- expression could promote melanoma cells to exit the blood and colonise lungs [22].

Colorectal carcinoma is one of the most common malignancies, with an increasing annual incidence [23]. Colorectal carcinoma is usually accompanied by local invasion and distant metastasis, which are the main causative factors for the cancer-related death [24]. However, the underlying molecular and cellular mechanisms are poorly understood. Our previous clinical study demonstrated that the levels of RhoA and RhoC mRNA transcripts in tumor tissues were significantly higher than those in the corresponding paratumor and normal tissues, and the expressions of both RhoA and RhoC in cancers with lymph node or liver metastasis were significantly higher than those in those without metastasis, indicating these two genes may contribute to the onset and development as well as invasion and metastasis of colorectal carcinoma. Specifically, the levels of RhoC expression were significantly correlated with the extents of local intestinal invasion although not with the histopathological degrees of cancers, strongly supporting its function in tumor invasion and metastasis [9]. Therefore, specific inhibitors of individual Rho functions are predicted to be of great therapeutic benefits.

RNA interference (RNAi) is an evolutionarily conserved sequence-specific post-transcriptional gene silencing mechanism triggered by small double-stranded RNA (dsRNA) that results either in degradation of homologues mRNAs or inhibition of mRNA translation [25]. Many studies have been done in down-regulating the expression of RhoA and RhoC by RhoA or RhoC-specific siRNAs to inhibit the proliferation and invasiveness of cancer cells [7,26,27]. In this study, we constructed an efficient multiple shRNAs expression system based on pSilence plasmid and our selected efficient two RhoA-targeted short hairpin RNAs (shRNA) and two RhoC-targeted shRNAs [8]. The tandem array multiple shRNAs expression vector contained four shRNA expression cassettes targeting two genes. In HCT116 cells, the multiple shRNAs expression constructs could efficiently and simultaneously induce inhibition of RhoA and RhoC genes and markedly inhibit the invasion and migration potentials of cancer cells. The inhibitory effects of multiple shRNAs expression vectors were more effective than single shRNA expression vector (data not shown). Further research work is being done to evaluate the inhibition effects of multiple shRNAs expression vectors on nude mice.

To our knowledge, this is the first study that 4-tandem shRNA construct targeting RhoA and RhoC genes was proved to be a successful approach in reducing the malignance of colorectal tumor cells. Recent accumulating evidences have shown that co-expression of multiple shRNAs can simultaneously inhibit multiple genes or target multiple sites on a single gene, which demonstrated that multiple shRNAs expression system could inhibit all six genes and was much more efficient in inducing apoptosis in PC3 cells [28]. Moreover, a tandem Ku-shRNA-encoding plasmid expression system can knock-down Ku70 and Ku80 at the same time [29]. Furthermore, the vector that expresses five shRNAs targeting on rat ventricular myocyte Kir2.1 gene in tandem is able to suppress the expression of Kir2.1 in rat ventricular myocytes [30]. All these results including ours implicate that such shRNA-induced in tandem RNA interference may be used for dissecting complex signaling pathways and even be applied to targeting multiple genes in cancer therapy.

## Competing interests

The authors declare that they have no competing interests.

## Authors' contributions

LXP, WHB and QS designed the research. LXP and WHB carried out the molecular genetic studies. SAH and SQ participated in the cell culture. YK and SQ discussed the results and analyzed data. LXP and WHB wrote the paper. All authors read and approved the final manuscript.
